# Brodie's abscess in a patient presenting with postchemotherapy neutropenic sepsis

**DOI:** 10.1002/jha2.199

**Published:** 2021-05-13

**Authors:** Lakmali Kandabadage, Eranga Perera, Priyankara Jayawardana, Abdeali Alibhoy, Jayantha Balawardena, Saman Hewamana

**Affiliations:** ^1^ Clinical Haematology Unit Lanka Hospitals Colombo Sri Lanka; ^2^ Lanka Hospital Colombo Sri Lanka; ^3^ National Hospital of Sri Lanka Colombo Sri Lanka; ^4^ Sir John Kotelawala Defence University Colombo Sri Lanka

An 18‐year‐old boy was diagnosed with diffuse large B‐cell lymphoma in the brain on November 28, 2020. He received combination chemotherapy cycle 1 on November 28, 2020 and cycle 2 on December 21, 2020. He was admitted with 4 days history of painful right wrist joint on December 12, 2020. On admission he was on Levatrazipam, Acyclovir, Pantoprazole, Fluconazole, Sucralfate and Desmopressin nasal spray.

On examination he had swollen, tender right wrist with restricted movements. His temperature was 38.5^°^C.

Laboratory investigations revealed Hb 75 g / L, white blood cell count 0.54 × 10^9^/L, neutrophils 0.11 × 10^9^/L, platelets 58 × 10^9^/L, ESR 117 mm/H, CRP 85.3 mg /L, and MRI revealed Brodie's abscess of the distal metaphysis radius.

He was started on piperacillin/tazobactam, amikacin and vancomycin intravenous; pain and swelling gradually subsided over a week and he was discharged on oral ciprofloxacin, cephalexin, and clindamycin to be continues for 6 weeks as per microbiology advice.

**FIGURE 1:**
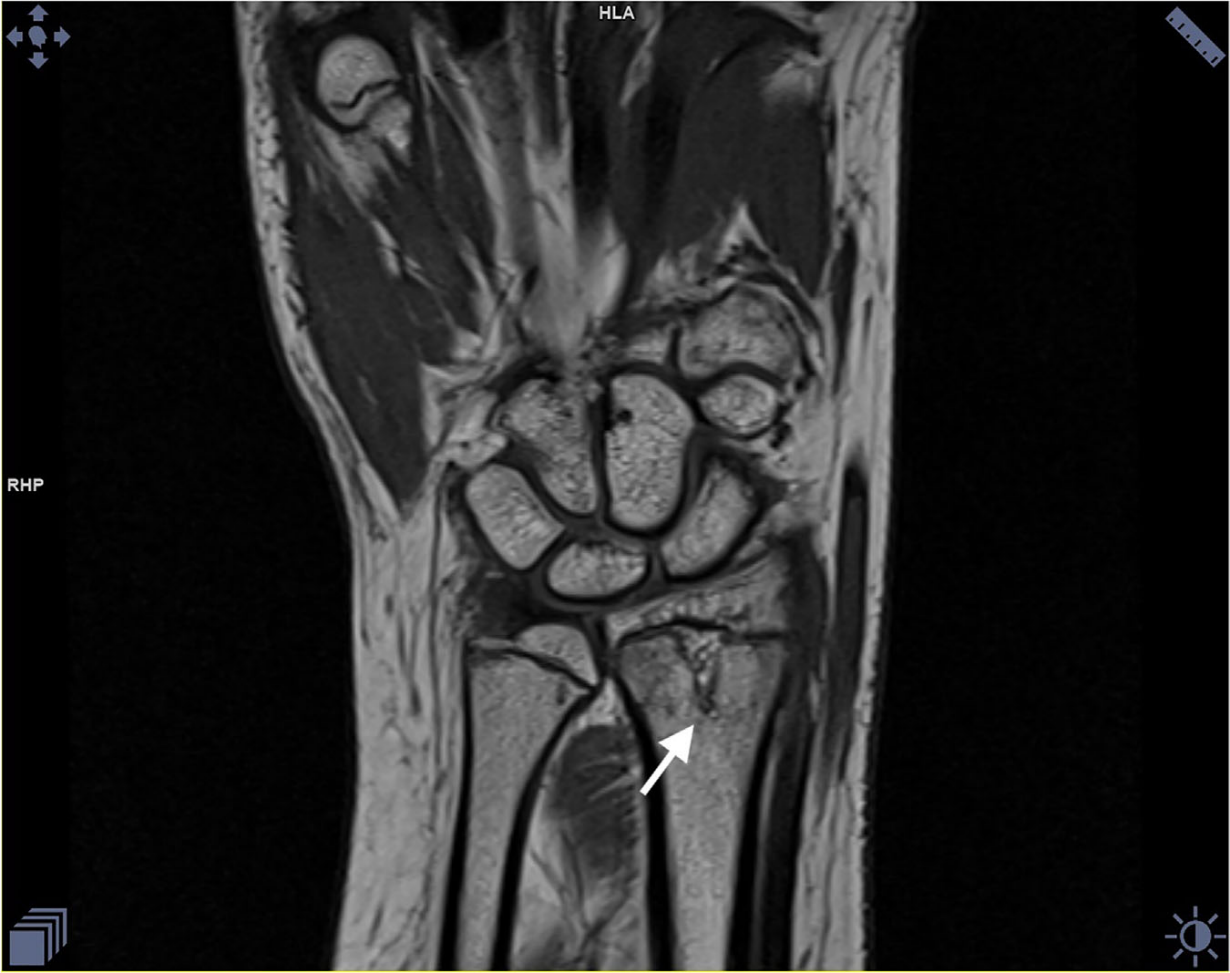
Coronal section of wrist shows a well‐defined finger‐like extension toward the epiphyseal plate with subtle marrow oedema (white arrow).

